# Thermal Inactivation of a Cold-Active Esterase PMGL3 Isolated from the Permafrost Metagenomic Library

**DOI:** 10.3390/biom9120880

**Published:** 2019-12-16

**Authors:** M.V. Kryukova, L.E. Petrovskaya, E.A. Kryukova, G.Yu. Lomakina, S.A. Yakimov, E.G. Maksimov, K.M. Boyko, V.O. Popov, D.A. Dolgikh, M.P. Kirpichnikov

**Affiliations:** 1 Kurchatov Complex of NBICS-technologies, National Research Centre “Kurchatov Institute”, Akad. Kurchatova sqr 1, 123182 Moscow, Russia; 2 Shemyakin and Ovchinnikov Institute of Bioorganic Chemistry, Russian Academy of Sciences, ul. Miklukho-Maklaya, 16/10, 117997 Moscow, Russia; 3 Department of Chemistry, Lomonosov Moscow State University, Leninskiye Gory, 1/3, 119991 Moscow, Russia; lomakinagalina@yahoo.com; 4 Department of Biology, Lomonosov Moscow State University, Leninskie gory, 1, bld 12, 119234 Moscow, Russia; emaksimoff@yandex.ru; 5 Bach Institute of Biochemistry, Research Center of Biotechnology of the Russian Academy of Sciences, Leninsky Ave. 33, bld. 2, 119071 Moscow, Russia; boiko_konstantin@inbi.ras.ru

**Keywords:** PMGL3 esterase, HSL family, thermal inactivation, cysteine mutagenesis, cold-active proteins, stability, hydrophobicity

## Abstract

PMGL3 is a cold-adapted esterase which was recently isolated from the permafrost metagenomic library. It exhibits maximum activity at 30 °C and low stability at elevated temperatures (40 °C and higher). Sequence alignment has revealed that PMGL3 is a member of the hormone-sensitive lipase (HSL) family. In this work, we demonstrated that incubation at 40 °C led to the inactivation of the enzyme (*t*_1/2_ = 36 min), which was accompanied by the formation of tetramers and higher molecular weight aggregates. In order to increase the thermal stability of PMGL3, its two cysteines Cys49 and Cys207 were substituted by the hydrophobic residues, which are found at the corresponding positions of thermostable esterases from the HSL family. One of the obtained mutants, C207F, possessed improved stability at 40 °C (*t*_1/2_ = 169 min) and increased surface hydrophobicity, whereas C49V was less stable in comparison with the wild type PMGL3. Both mutants exhibited reduced values of *V_max_* and *k_cat_*, while C207F demonstrated increased affinity to the substrate, and improved catalytic efficiency.

## 1. Introduction

Cold-active enzymes represent a promising resource for biotechnological applications since they potentially allow avoiding energy loss for the heating of the reaction mixture and inactivation of the heat-labile compounds [1,2,3,4,5]. Therefore, the rapidly growing number of the cold-active enzymes has been obtained to date using the available genomic sequences of the cold-adapted microorganisms and metagenomic approaches [6,7].

Many cold-active enzymes demonstrate attractive catalytic properties (high catalytic efficiency and activity at low temperatures). However, their usage is often limited by low thermal stability [8,9]. Most psychrophilic enzymes usually possess a half-life about 10 min at 50–60 °C and unfold rapidly at high temperatures or at other denaturating conditions. Loss of the activity upon storage also represents a fundamental obstacle to the wider utilization of the cold-active enzymes. These undesirable effects are explained by the increased conformational mobility of their molecules, which provides the ability to perform reactions at low temperatures [10,11]. Structural features contributing to the low thermal stability of cold-adapted proteins can generally be described as a decreased number of stabilizing interactions in their molecules, including smaller hydrophobic core, a smaller number of salt bridges, and the presence of long extended loops with lower numbers of proline residues in comparison with the homologs from mesophilic and thermophilic microorganisms. In addition, the surfaces of cold-active enzymes contain more hydrophobic residues [2,8,12].

To obtain the improved biocatalysts which combine high activity at low temperatures and thermal stability, numerous protein engineering approaches have been developed and employed [13,14]. In many cases, random mutagenesis provides a suitable solution to the problem. However, the determination of the specific structural mechanism of inactivation opens an opportunity to concentrate efforts on the particular site of the molecule and to minimize loss of the resources.

Cold-active lipolytic enzymes catalyze the cleavage of the ester bond in triacylglycerols and are classified into lipases and esterases depending on the length of the acyl chain of the preferred substrate [15]. They can be used in the food industry, biodiesel, and detergent production, fine chemical synthesis etc. [16,17]. The molecules of the lipases and esterases consist of a lid domain and a catalytic domain, and the latter possesses a typical α/β hydrolase fold [18,19]. The catalytic triad consists of Ser(nucleophil)-Asp/Glu-His. Lipolytic enzymes belonging to the hormone-sensitive lipase (HSL) family are widely represented in different classes of organisms and frequently isolated as a result of metagenomic studies [20]. Their amino acid sequences are characterized by the presence of conserved sequence motives HGGGF and GXSXG, the latter including a catalytic serine residue [21].

In this work, we describe thermal inactivation of the cold-active esterase PMGL3, which is a member of the HSL family. This enzyme was isolated from a metagenomic library obtained from a Siberian permafrost sample. It demonstrated typical cold-adapted behavior, including a low-temperature optimum (30 °C) and low stability at elevated temperatures [22]. In the current study, we have shown that the thermal inactivation of PMGL3 is accompanied by the formation of oligomers and high molecular weight aggregates. The PMGL3 molecule contains two cysteine residues, which frequently represent the sites for undesired oxidation and subsequent activity loss of proteins [23,24]. In order to eliminate the possible source of instability and to increase hydrophobic interactions in PMGL3, we introduced substitutions C49V and C207F and characterized the obtained mutant variants. It was demonstrated that the point mutation C207F results in increased thermal stability of the mutant protein and reduced its tendency to oligomerization, while the other mutant, C49V, was less stable in comparison with wild type PMGL3.

## 2. Materials and Methods

Reagents from Bio-Rad (Hercules, California, USA), Merck (Darmstadt, Germany), Panreac (Barcelona, Spain), and organic solvents from Chimmed (Moscow, Russia) were used in the study. *p*-nitrophenyl ester substrates and bis-ANS were obtained from Sigma (St. Louis, MO, USA). Solutions were prepared using MilliQ water (Millipore, Darmstadt, Germany). Primers were synthesized by Evrogen (Moscow, Russia).

### 2.1. Gene Cloning and Mutant Construction

DNA manipulations were performed by standard methods using enzymes from Thermo Fisher Scientific (Waltham, MA, USA). The mutant genes were constructed by two-step SOE (splicing with overlap extension)-PCR using wild-type PMGL3 gene as a template with *Pfu* DNA polymerase, and primers C49V for CCGAGGGCGTTTTGATCGAGC and C49Vrev GCTCGATCAAAACGCCCTCGG for the C49V mutant; C207F for CTGACCGGATTCTCGGCG and C207Frev CCGCCGAGAATCCGGTCAG for the C207F mutant. The resulting products were cloned into the pET32a vector, as described in [22]. Mutations were confirmed by DNA sequencing (Evrogen).

### 2.2. Protein Purification

*Escherichia coli* BL21(DE3) cells transformed with the recombinant plasmids were grown at 37 °C and 250 rpm in 50 mL of LB medium supplemented with ampicillin (100 μg/mL). After the cell culture optical density reached 0.6–0.8 at 560 nm, expression was induced with 0.1 mM isopropyl β- D-1-thiogalactopyranoside (IPTG). After incubation at 22 °C and 200 rpm for 4 h, induced cells were harvested by centrifugation (4000× *g*, 15 min at 4 °C) and resuspended in buffer A (20 mM Tris-HCl, 200 mM NaCl, pH 8.0, 10 mM 2-mercaptoethanol). The cell suspension was sonicated using a Branson Sonifier 450 on an ice bath for 5 min (15 sec on, 1 min off). Cell debris was removed by centrifugation at 16,000× *g* for 10 min. The supernatant was applied to a disposable column with 1 mL of Ni-Sepharose Fast Flow resin (GE Healthcare) equilibrated with buffer A containing 10 mM imidazole. The column was extensively washed with buffer A containing 500 mM NaCl and 20 mM imidazole. Bound proteins were eluted with buffer B (20 mM Tris-HCl, 200 mM NaCl, 100 mM imidazole, pH 8.0). Samples were analyzed by SDS-PAGE with a 13% separating gel. Combined fractions containing the target protein with purity more than 90% were dialyzed against buffer C (20 mM Tris-HCl pH 8.0, 150 mM NaCl, 10 mM 2-mercaptoethanol) and sterilized by filtration. Protein concentration was measured with a Protein Assay Kit (Bio-Rad) using BSA as a standard. The yield was about 5 mg of protein from 50 mL of medium.

### 2.3. Esterase Assay

Esterase activity assay was performed using *p*-nitrophenyl butyrate (*p*-NPB) as a substrate in 96-well assay plates (Deltalab, Barcelona, Spain). Purified PMGL3 or mutant variants (2 μg) were added to 1 mL of the reaction mixture containing 50 mM Tris HCl pH 8.0, 100 mM NaCl, and 0.25 mM *p*-NPB. Each unit of activity was determined as an amount of enzyme releasing 1 μmol of *p*-nitrophenol per minute. Kinetic parameters of the enzyme reaction were determined from the Michaelis–Menten equation using *p*-NPB substrate concentrations from 0.1 to 1.25 mM. The values for *K_m_* and *V_max_* were estimated by linear regression from a Lineweaver–Burk plot (1/V_0_ from 1/S). The optimum reaction temperature was determined by measuring the esterase activity at different temperatures (5–50 °C). The reaction mixture was incubated at each temperature for 10 min without enzyme and substrate and after their addition activity was measured as described above. The thermal stability of the enzymes was determined by measuring residual activity under standard conditions after incubation at different temperatures from 5 to 50 °C. The half-time of inactivation, *t*_1/2_, was calculated from the time-course study as first-order kinetics.

### 2.4. Circular Dichroism Spectra

Circular dichroism (CD) spectra were recorded on a Chirascan CD spectrometer (Applied Photophysics, Leatherhead, UK) at 180–320 nm at a constant time of 3 s, scan speed 10 nm/min. Spectra were measured at different temperatures (20–50 °C) in the protein samples with a concentration of 0.6 mg/mL in 20 mM K_2_PO_4_, and 150 mM KF. The content of the secondary structure elements was calculated with the DichroWeb service (http://pcddb.cryst.bbk.ac.uk) using the CDSSTR method and reference data sets 4, 7, SMP180.

### 2.5. Size-exclusion Chromatography

Size exclusion chromatography (SEC) of purified proteins was conducted on a Superdex 75 10/300 GL column (GE Healthcare, Chicago, Illinois, USA) at a flow rate of 0.4 mL/min in 100 mM Tris-HCl, pH 8.0, and 150 mM NaCl.

### 2.6. Fluorescence Measurements

Fluorescence decay kinetics with a picosecond time resolution of tryptophan residues and bis-ANS (4,4′-Dianilino-1,1′-Binaphthyl-5,5′-Disulfonic Acid) were collected by time- and wavelength-correlated single-photon counting setup based on an HMP-100-07C detector and SPC-150 module (Becker and Hickl, Berlin, Germany). Protein concentration was 0.55 mg/mL in 50 mM HEPES, and 150 mM NaCl (pH 7.5). Fluorescence excitation was performed by Yb femtosecond laser TEMA-150 (Avesta, Moscow, Russia), driven at 80 MHz repetition rate, delivering 150 fs pulses to the sample at 262.5 nm (4th harmonics). Fluorescence decay was approximated by a sum of three exponential decay functions with the SPCImage software package (Becker and Hickl, Berlin, Germany), considering the incomplete decay components with long lifetimes. To compare different decay curves, we calculated the average decay time according to the following expression: $\tau_{av}=\sum_{i}^{n}\tau_{i}a_{i}$, where $\tau_{i}$ and $a_{i}$ are the lifetime and the amplitude (normalized to unity: $\sum_{i}^{n}a_{i}=1$) of the i-th fluorescence decay component, respectively. The temperature of the sample was controlled by a Qpod 2e (Quantum Northwest, Liberty Lake, WA, USA) cuvette holder. Fluorescence decay kinetics were measured upon the temperature-induced unfolding of proteins, which was induced by heating at a constant rate (1 °C per minute) from 20 to 80 °C [25]. Approximation of the experimental data, i.e., the temperature dependence of normalized parameters of the decay of tryptophan residues or bis-ANS fluorescence in proteins (Figure S2), was performed using a Boltzmann-type equation as described in [26] after linearization of the temperature dependence, considering that the initial slope at low temperatures represents a stable protein state while the following sigmoid section was due to unfolding (Figure S3). This approach allowed us to determine melting temperatures (*T_m_*) in order to estimate protein stability. Each experiment was conducted at least three times.

### 2.7. Bioinformatic Analysis

Multiple protein alignment was produced with Clustal Omega (https://www.ebi.ac.uk/Tools/msa/clustalo/) and visualized with Jalview v2.9.0. A structure model of PMGL3 was generated using the SWISS-MODEL server based on Protein Data Bank entry 5GMR as a template and drawn using Swiss-PdbViewer.

## 3. Results

### 3.1. Thermal Inactivation of PMGL3 is Accompanied by Oligomerization

Previously, we have expressed a cold-active esterase PMGL3 from a permafrost metagenomic library and demonstrated that it possessed maximum activity at 30 °C with *p*-nitrophenyl butyrate (C4) as a substrate. PMGL3 demonstrated low thermal stability as incubation for 60 min at 50 °C led to its complete inactivation [22].

**Figure 1:**
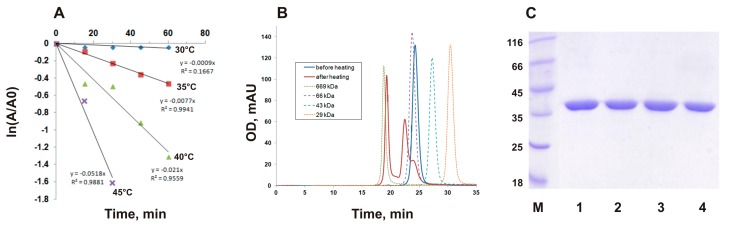
Thermal inactivation of cold-adapted esterase, PMGL3. (**A**) Thermal inactivation profile of wt PMGL3 at different temperatures. The enzyme was incubated at indicated temperatures, and the samples were taken for esterase activity assay at 30 °C at regular time intervals. (**B**) Gel filtration analysis of PMGL3 (fresh sample and after incubation for 1 h at 40 °C) was performed on a Sephadex G-75 column calibrated by carbonic anhydrase (29 kDa), ovalbumin (43 kDa), BSA (66 kDa), and thyroglobulin (669 kDa). (**C**) SDS-PAGE of wt PMGL3 before (lines 1, 2) and after (lines 3, 4) incubated for 1 h at 40 °C. Lines 1, 3—sample buffer containing β-mercaptoethanol; lines 2, 4—sample buffer without β-ME. M—protein molecular weight markers (Thermo Fisher).

In the current work, we performed a detailed study of the PMGL3 thermoinactivation in a temperature range of 30–45 °C (Figure 1A). Analysis of the residual activity of PMGL3 after incubation at different temperatures demonstrated that it was stable at 30 °C (*t*_1/2_ 770 min), while at 40 and 45 °C, thermoinactivation of the enzyme was observed (*t*_1/2_ 36 and 13 min, correspondingly, Figure 1A).

To further describe this process, we performed gel filtration analysis of PMGL3 before and after heat treatment (Figure 1B). It has shown that the protein before heating elutes from the size exclusion column as a single peak, with the mobility corresponding to a dimeric form (molecular weight ~70 kDa). Upon incubation for 1 h at 40 °C, the appearance of a second peak was observed, which corresponds to a tetrameric form of the protein. However, the major fraction of the sample was represented by the multimeric species with molecular weight ~600 kDa. Consequently, we can conclude that incubation at elevated temperatures (40 °C and higher) led to aggregation and simultaneous inactivation of PMGL3.

### 3.2. Disulfide Bond Formation does not Contribute to Oligomerization of PMGL3

The PMGL3 contains two cysteine residues, Cys49 and Cys207. According to the results of the homology modeling of this protein (Figure S1), they are situated at the opposite sides of the molecule, and thus, are reasonably likely not connected by a disulfide bond. However, there was a possibility of intermolecular oxidation of these residues in the course of the heat treatment, which may potentially result in aggregation. To verify this hypothesis, we performed an electrophoretic analysis of PMGL3 before and after incubation at 40 °C in the absence of β-mercaptoethanol in the sample buffer. Figure 1C demonstrates that the electrophoretic mobility of PMGL3 corresponds to a monomer protein, irrespectively of the presence of a reducing agent. We have concluded that intermolecular disulfide bond formation is not the reason for heat-induced aggregation and inactivation of PMGL3 in the applied conditions. However, we could not exclude the possibility of cysteine oxidation in PMGL3 as a result of prolonged incubation at higher temperatures or in the presence of the reactive substances in the medium. Therefore, we decided to study the effects of amino acid substitutions of cysteine residues on the thermal stability of PMGL3.

### 3.3. Construction and Properties of the Mutant Proteins C49V and C207F

**Figure 2:**
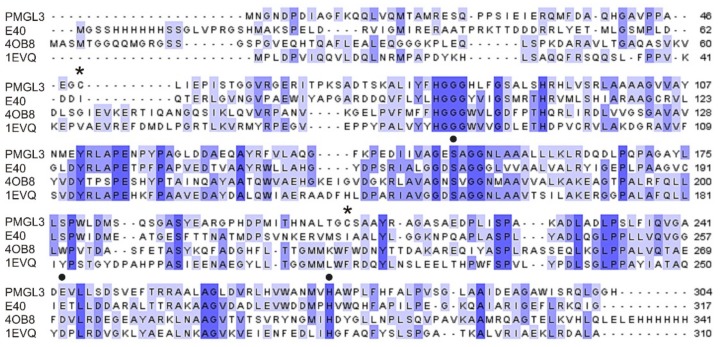
Multiple sequence alignment of PMGL3 and esterases with the highest similarity from the hormone-sensitive lipase (HSL) family using Clustal Omega. The extent of amino acid sequence conservation is depicted in grades of blue. Black circles mark amino acid residues belonging to the catalytic triad, and the asterisks indicate the position of cysteine residues in PMGL3. E40—thermolabile esterase E40 from a marine sedimental metagenomic library [27]; 4OB8—esterase from *Pseudomonas putida* ECU1011 [28]; 1EVQ—thermostable esterase Est2 from *Alicyclobacillus acidocaldarius* [29].

Alignment with homologous proteins, including enzymes from thermophilic microorganisms (Figure 2), revealed that at the positions corresponding to Cys49 and Cys207 of PMGL3 the hydrophobic residues are usually found. Recently, Li et al. [27] compared the structures of thermophilic HSL lipases and their mesophilic homologs and demonstrated that intra- and interdomain hydrophobic interactions represent a significant contribution to the stability of the thermophilic members of this family including rPPE esterase from *Pseudomonas putida* [28], and Est2 from *Alicyclobacillus acidocaldarius* [29]. Correspondingly, the thermal stability of a thermolabile esterase, E40, from a marine sedimental metagenomic library was significantly improved by the introduction of two adjacent aromatic residues, which are conserved in thermostable HSLs (mutations S202W and I203F). It should be mentioned that the I203F mutant demonstrated increased stability in comparison with S202W that points to the greater importance of the phenylalanine residue in the stabilization of the E40 protein structure. Similarly to E40, in the corresponding regions of PMGL3, the hydrophobic residues were absent (Figure 2). We decided to substitute Cys49 for the valine and Cys207 for the phenylalanine residues, which are present at the corresponding positions in the amino acid sequence of several thermostable esterases from the HSL family [23].

**Figure 3:**
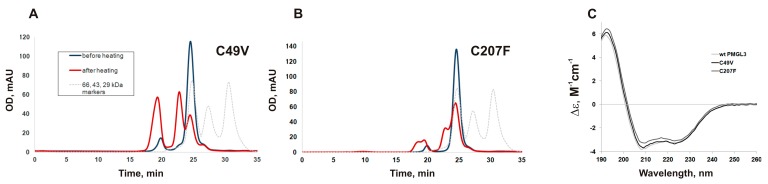
Properties of the mutant variants C49V and C207F. Gel filtration analysis of PMGL3 mutants C49V (**A**) and C207F (**B**) on a Sephadex G-75 column before and after incubation for 1 h at 40 °C. (**C**) Far-UV circular dichroism (CD) spectra of wt PMGL3 and its mutant variants at pH 8.0.

Mutant variants of PMGL3 C49V and C207F were constructed by site-directed mutagenesis and produced in *E. coli* cells. SEC analysis demonstrated that the mutants formed dimers in solution with the chromatographic mobility similar to the wild-type protein (Figure 3A,B). Their temperature optimum was equal to that of PMGL3 (30 °C). To compare the thermal stability of the mutants with that of PMGL3, we have measured their half-life times upon incubation at 40 °C (Table 1). According to these data, the rate of inactivation of the C49V mutant was even higher than that of the wild type (*t*_1/2_ 27 min), while the C207F mutation resulted in a significant increase in stability (*t*_1/2_ 169 min).

We also performed a gel filtration analysis of the mutants after their incubation for 1 h at 40 °C. Similar to the wt PMGL3, the mutant proteins C49V and C207F demonstrated a transition from a dimeric state to a tetramer and high molecular weight aggregates as a result of the treatment, which additionally demonstrated a vague role of cysteines in the process of aggregation. The chromatographic profile of C49V was very similar to the wild type protein. However, the amount of the dimer left in preparation after heating was higher in comparison with the wild type protein (Figure 3A). According to these results, the C207F protein sample contained the least amount of aggregates and the highest proportion of the native dimer (Figure 3B).

CD measurements of PMGL3, C49V, and C207F at 20 °C demonstrated the typical spectra of the α/β hydrolase family members with a large percentage of irregular structure, which is characteristic for the cold-adapted proteins (Figure 3C and Table S1). Analysis of the data with DichroWeb service revealed small changes in the secondary structures of the C49V and C207F proteins in comparison with the wild type PMGL3, including the increased content of the beta-sheets in the mutants (Table S1).

**Figure 4:**
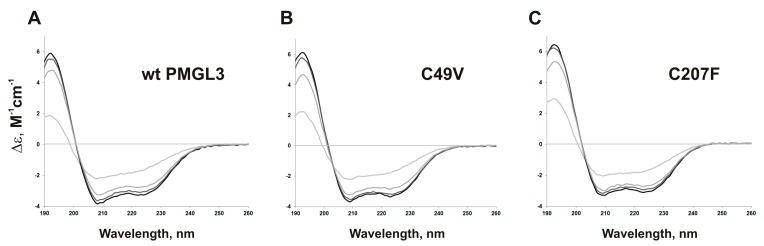
CD spectra in the 190- to 260-nm range of the wild type PMGL3 (**A**), C49V (**B**), and C207F (**C**) mutants. Spectra were recorded from 20 °C (black line) to 50 °C (light grey line).

To reveal the structural changes associated with the increase of temperature, we performed additional CD experiments with PMGL3 and its mutant variants at 30, 40, and 50 °C. All three proteins underwent a similar large spectral change upon transition from 40 to 50 °C, presumably reflecting the unfolding of their molecules (Figure 4). However, it can be seen that the content of alpha-helices and beta-sheets in C207F mutant was almost constant upon heating from 20 to 40 °C, while it became significantly reduced at 50 °C. In contrast, the number of alpha-helices in the wild type PMGL3 and the C49V mutant already decreased at 40 °C at 4 and 5% correspondingly. In addition, the amount of irregular structure was minimal in C207F in comparison with PMGL3 and C49V (Table S1). Thus, the secondary structure of the C49V mutant displayed decreased thermal stability in these experiments, while it was more stable in the C207F mutant that is in line with the results of the thermoinactivation measurements and fluorescence studies (see below).

The kinetic parameters for the wild type PMGL3 and its mutant variants were obtained with C4 as a substrate at 30 °C (Table 1). We have shown that the catalytic properties of the esterase were significantly affected by mutations. The affinity to the substrate was reduced by a factor of 1.3 in C49V, while in the C207F mutant, it increased by 5.8-fold. *V_max_* was reduced in both mutants by a factor of 1.74. The catalytic constant *k_cat_* was similar for both mutants and decreased by 1.74-fold in comparison with the wild type. Remarkably, the catalytic efficiency of the C207F mutant increased by 3.3-fold in comparison with PMGL3 while it was reduced by 2.3-fold for C49V.

### 3.4. bis-ANS Binding Studies of PMGL3 and Mutant Variants

**Figure 5:**
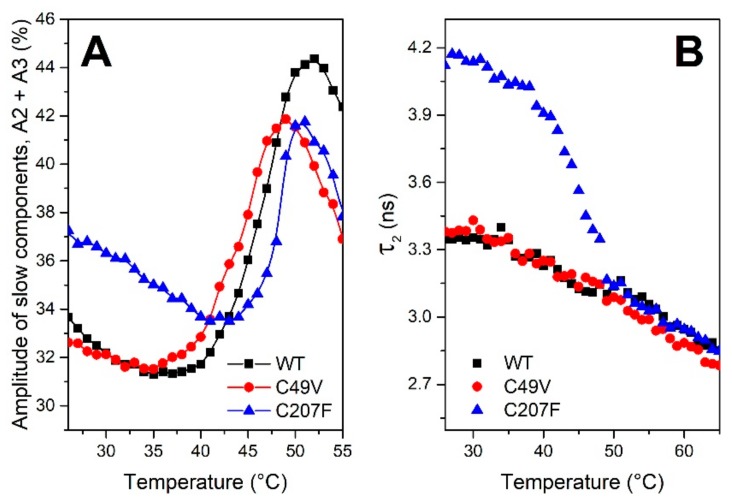
Temperature dependence of fluorescence emission intensity of bis-ANS (4,4′-Dianilino-1,1′-Binaphthyl-5,5′-Disulfonic Acid) in the presence of PMGL3 and mutant variants. (**A**) Amplitude of the middle and slow components (A2 + A3) of the fluorescence decay kinetics of bis-ANS associated with dye binding to the hydrophobic sites of the proteins. (**B**) Lifetime of the middle component τ_2_ of bis-ANS fluorescence in the protein solutions. Parameters for both types of temperature dependencies were estimated upon approximation of the fluorescence decay curves (Figure S2) by the sum of three exponential decay functions (see Methods). The dye to protein ratio was 10:1 in all experiments.

A hydrophobic dye bis-ANS is widely used for quantification of the protein hydrophobicity [30]. Fluorescence of bis-ANS in aqueous solutions is characterized by a short lifetime (~160 ps). The addition of a protein causes a dramatic increase of bis-ANS fluorescence intensity, quantum yield, and lifetime. This effect is explained by the availability of protein hydrophobic sites at which bis-ANS is isolated from interactions with the solvent [30]. In excess of the dye, its fluorescence is characterized by non-monoexponential decay due to the different environments in which bis-ANS is present (Figure S2). Time-resolved measurements of bis-ANS fluorescence in the presence of equal concentrations of PMGL3 and mutant proteins C49V and C207F revealed that the number of the hydrophobic sites (estimated as the total yield of slow components of fluorescence decay) was increased in the C207F mutant in comparison to those of PMGL3 and C49V (Figure 5A). Correspondingly, the lifetimes of the slow components were higher in the C207F mutant (Figure 5B). These data indicate that not only was the number of hydrophobic sites increased, but also the exposure of dye to the solvent in these sites decreased in comparison with the wild type PMGL3 and the C49V mutant.

In all cases, fluorescence intensity and quantum yield of bis-ANS in protein solutions decreased almost linearly with the increase of the temperature from 20 to 35 °C, which is an indicator of the protein structural stability (Figure 5). However, further increase of the temperature caused significant growth of the yield of the slow components of fluorescence decay (A2 + A3), which we assume to be associated with the unfolding of the proteins and exposure of the novel hydrophobic sites.

After linearization of the temperature dependence, approximation of the experimental data by a Boltzmann-type equation allowed us to determine melting temperatures *T_m_* in order to compare the thermal stability of proteins. In agreement with observed changes in enzymatic activity, we have found that the stability of C49V was decreased, while C207F was more stable than the wild type PMGL3 (Figure S3 and Table 1).

## 4. Discussion

Increasing protein stability and avoiding aggregation are the fundamental issues for the development of efficient and robust biocatalysts for industrial applications [5,31]. Protein aggregation at elevated temperatures is usually explained by the partial unfolding of the protein molecules and concomitant interactions between newly exposed hydrophobic regions [32,33,34]. Due to particular structural and functional characteristics (ability to bind hydrophobic substrates and to operate in the conditions of low water activity), the lipolytic enzymes may possess an increased tendency to self-assemble and aggregate [35]. Furthermore, increased flexibility and exposed hydrophobic surfaces, which are characteristic for cold-adapted proteins can represent the basis for the enhanced aggregation potential of the cold-active lipases. Indeed, numerous cold-active lipases were reported to produce high molecular weight aggregates upon microbial production or thermal denaturation of isolated proteins [36,37,38,39]. In this study, we have observed that the cold-active and thermolabile esterase PMGL3 predominantly converted from the initial dimeric state into tetramers and higher molecular weight aggregates after incubation for 1 h at 40 °C. We can presume that the loss of conformation upon PMGL3 heating leads to exposure of the hydrophobic regions and their subsequent aggregation.

Several studies pointed to the cysteine interchange and formation of intermolecular disulfide bridges as a major factor contributing to aggregation [38,40]. Cysteine residues are also subject to chemical oxidation, which leads to a loss of activity [24]; therefore, amino acid substitutions at these positions can lead to increased stability [41,42,43]. By using non-reducing SDS-PAGE, we demonstrated that the two cysteine residues of PMGL3 apparently do not participate in disulfide bond formation, presumably due to their low solvent accessibility. However, prolonged incubation at elevated temperatures or in other harsh conditions can promote their oxidation.

To develop an efficient approach for the stabilization of the cold-active enzymes against thermal inactivation, sequence and structural comparisons with thermostable homologs are traditionally performed. Enzymes from thermophilic organisms are characterized by an increased number of intramolecular interactions including hydrogen bonds, hydrophobic, and electrostatic interactions [44,45,46,47,48]. Systematic analysis of the impact of different factors on the stability of a set of 373 thermophilic proteins and their mesophilic homologs demonstrated that the hydrophobic environment is a major factor that provides increased stability at elevated temperatures [46]. The role of hydrophobic residues in the stability of lipolytic enzymes was demonstrated by numerous studies. Mutations of the tryptophan residues belonging to the lid domain were shown to decrease thermostability of the lipases from *Bacillus thermocatenulatus* [49] and *Humicola lanuginosa* [50]. On the contrary, the introduction of additional hydrophobic residues into lipase T6 from *Geobacillus stearothermophilus* [51], *P. fragi* lipase PFL [52], esterase E40 [27], and lipase A from *Bacillus subtilis* [53] resulted in increased stability. In the current work, we introduced the phenylalanine residue at the position of Cys207 (mutation C207F). Previously, it was shown that mutation of the homologous residue I203F led to a more than 100-fold increase in the *t*_1/2_ of a thermolabile esterase E40 at 40 °C [27]. The second Cys49 was substituted for a valine residue, which is found in the corresponding positions in the molecules of the thermophilic esterases Est2 (1EVQ), Sto-Est (3AIK), and AFEst (1JJI) [27].

Studies of the thermal inactivation profiles of the obtained mutants demonstrated that the introduction of the hydrophobic residues at two different sites of the cold-active esterase PMGL3 resulted in opposite effects. The C207F mutation led to a 4.7-fold increase in the half-life time of the protein at 40 °C (Table 1). On the contrary, the C49V mutant demonstrated reduced stability in comparison with the wild type protein (*t*_1/2_ by 1.3-fold smaller than in PMGL3).

According to the results of bis-ANS binding studies, the C207F mutant demonstrated an increase in the number of the hydrophobic sites while in C49V, the amount of bound dye was equivalent to the wild type PMGL3 (Figure 5). Fluorescence measurements also allowed determination of the melting temperatures of PMGL3 and the mutants by two independent methods (bis-ANS binding and intrinsic fluorescence of tryptophan residues). The *T_m_* values were slightly lower for the intrinsic probe due to higher sensitivity of the tryptophan residues to early conformational rearrangements in protein structure, which eventually resulted in the exposure of novel hydrophobic sites involved in bis-ANS binding. However, the observed tendency was the same for both types of the probes, i.e., the stability of the C207F mutant was increased in comparison with the wild type protein while the unfolding of the C49V mutant occurred at a lower temperature (Table 1). It should be mentioned that the melting temperatures of PMGL3 and the mutants are typical for cold-adapted proteins. For example, the *T_m_* value of 45 °C was reported for the psychrophilic α-amylase from *Pseudoalteromonas haloplanktis* in comparison with 65 °C for the mesophilic pig pancreatic α-amylase [54]. The recently described cold-adapted lipase from *Pseudomonas sp.* LSK25 exhibited a *T_m_* = 47 °C [55]. The obtained results were consistent with the CD studies of the secondary structure of PMGL3 and the mutant proteins. All three proteins demonstrated a significant reduction in the alpha-helical content upon heating to 50 °C; however, the extent of its loss at 30 °C and 40 °C was smaller in the C207F mutant.

The mutant proteins demonstrated similar *V_max_* and *k_cat_* values, which were decreased in comparison with the wild type PMGL3. The high reaction rate of PMGL3 indicates that the enzyme is optimized for the catalytic activity at low temperatures. Due to the increased dynamics of the active site, the binding of the substrates to the cold-active enzymes occurs less tightly, resulting in higher *K_m_* [8,10]. In support of this tendency, *K_m_* of the less stable mutant C49V is increased by 1.3-fold in comparison with the wild type protein, while increased stability of the C207F mutant was accompanied by 5.8-fold higher affinity (smaller *K_m_*) to the substrate.

The dissimilar consequences of the cysteine mutations in PMGL3 could be explained, taking into account their different locations in the molecule. According to the model of PMGL3 structure (Figure S1), the Cys207 residue is located in α7 helix not far from the catalytic triad and the substrate-binding pocket of the enzyme. Recently, it was shown that in the molecule of the S202W I203F mutant of E40 esterase, the newly introduced phenylalanine residue protrudes into the substrate-binding pocket in a similar orientation to the isoleucine in the wild type protein [56]. However, the distance from the larger side chain of F203 to the surrounding residues was shorter that could strengthen the hydrophobic interactions in the catalytic domain. As a result, the mutant protein demonstrated increased thermal stability and a lower *K_m_* value in comparison with the wt E40 protein. It can be speculated that the presence of a bulky aromatic residue also rigidifies the configuration of the substrate-binding pocket of the C207F mutant of PMGL3, resulting in firmer binding of the substrate and lower *K_m_*. Increased hydrophobicity of this mutant presumably represents a prerequisite for its enhanced thermal stability. On the contrary, the introduction of the hydrophobic valine residue in the vicinity of the loop, which connects the cap and the catalytic domain of PMGL3 (Figure S1), may lead to destabilization of their interaction and enhanced flexibility of the substrate pocket in the C49V mutant. The important role of this loop in determining catalytic properties and stability of the enzymes belonging to the α/β hydrolase family was demonstrated [57]. Undoubtedly, an appropriate interpretation of the obtained results requires the structure determination of PMGL3 and its mutant variants, which is planned for future studies.

## 5. Conclusions

In summary, we provided a detailed characterization of the thermal inactivation profile of the cold-active esterase PMGL3 and established its connection with the formation of high molecular weight aggregates. The introduction of the phenylalanine residue in the C207F mutant variant promoted an increase in the thermal stability of the protein. The mutation also affected the hydrophobicity and catalytic properties of PMGL3. As a result, our study produced additional data on the significance of protein engineering approaches for increasing the thermal stability of HSL family enzymes.

## Figures and Tables

**Table 1:** Characteristics of PMGL3 and the mutant variants.

Protein	*t*_1/2_, min (40 °C)	*T_m_*_,_ °C	*K_m_,* mM	*k_cat_,* min^−1^	*V_max_,* mM/min/mg	*k_cat_*/*K_m_,* min^−1^ mM^−1^
**PMGL3**	35.7	46.1 ^a^/40.1 ^b^	0.98	5490	164.7	5602
**C49V**	27.4	43.6 ^a^/36.9 ^b^	1.29	3156	94.7	2446
**C207F**	169.1	47.8 ^a^/43.4 ^b^	0.17	3152	94.6	18,541

^a^—melting temperature (*T_m_)* was determined by the fluorescence of bis-ANS. ^b^—*T_m_* was determined by the fluorescence of Trp residues.

## Supplementary Materials

The following are available online at https://www.mdpi.com/2218-273X/9/12/880/s1, Figure S1: Location of cysteine residues and residues belonging to the catalytic triad in the PMGL3 molecule, Figure S2: Characteristic fluorescence decay kinetics of bis-ANS in the buffer and in protein solutions of PMGL3 and mutant variants at 25 °C, Figure S3: Melting of PMGL3 and mutant variants as revealed by bis-ANS fluorescence (A) and intrinsic Trp fluorescence (B), Table S1: Content of the secondary structure elements in PMGL3 and mutant variants obtained by CD spectroscopy at different temperatures.

## References

[1] Margesin R., Feller G., Gerday C., Russell N.J. (2002). Cold-adapted microorganisms: Adaptation strategies and biotechnological potential. Encycl. Environ. Microbiol..

[2] D’Amico S., Claverie P., Collins T., Georlette D., Gratia E., Hoyoux A., Meuwis M.-A., Feller G., Gerday C. (2002). Molecular basis of cold adaptation. Philos. Trans. R. Soc. London.

[3] Cavicchioli R., Siddiqui K.S., Andrews D., Sowers K.R. (2002). Low-temperature extremophiles and their applications. Curr. Opin. Biotechnol..

[4] Littlechild J.A. (2015). Enzymes from extreme environments and their industrial applications. Front. Bioeng. Biotechnol..

[5] Barroca M., Santos G., Gerday C., Collins T., Margesin R. (2017). Biotechnological Aspects of Cold-Active Enzymes. Psychrophiles: From Biodiversity to Biotechnology.

[6] Santiago M., Ramírez-Sarmiento C.A., Zamora R.A., Parra L.P. (2016). Discovery, Molecular Mechanisms, and Industrial Applications of Cold-Active Enzymes. Front. Microbiol..

[7] Ferrer M., Martínez-Martínez M., Bargiela R., Streit W.R., Golyshina O.V., Golyshin P.N. (2016). Estimating the success of enzyme bioprospecting through metagenomics: Current status and future trends. Microb. Biotechnol..

[8] Siddiqui K.S., Cavicchioli R. (2006). Cold-adapted enzymes. Annu. Rev. Biochem..

[9] Feller G., Gerday C. (2003). Psychrophilic enzymes: Hot topics in cold adaptation. Nat. Rev. Microbiol..

[10] Feller G. (2013). Psychrophilic enzymes: From folding to function and biotechnology. Scientifica.

[11] Elleuche S., Schröder C., Sahm K., Antranikian G. (2014). Extremozymes—biocatalysts with unique properties from extremophilic microorganisms. Curr. Opin. Biotechnol..

[12] Kovacic F., Mandrysch A., Poojari C., Strodel B., Jaeger K.-E. (2016). Structural features determining thermal adaptation of esterases. Protein Eng..

[13] Bornscheuer U.T., Kourist R., Lee S.Y. (2017). Evolving Enzymes for Biocatalysis. Consequences of Microbial Interactions with Hydrocarbons, Oils, and Lipids: Production of Fuels and Chemicals.

[14] Kourist R., Brundiek H., Bornscheuer U.T. (2010). Protein engineering and discovery of lipases. Eur. J. Lipid Sci. Technol..

[15] Tutino M.L., Parrilli E., Santi C., Giuliani M., Marino G., Pascale D. (2010). Cold-adapted esterases and lipases: A biodiversity still under-exploited. Curr. Chem. Biol..

[16] Romano D., Bonomi F., Mattos M.C., Fonseca T.D., Oliveira M.D.F., Molinari F. (2015). Esterases as stereoselective biocatalysts. Biotechnol. Adv..

[17] Joseph B., Ramteke P.W., Thomas G., Shrivastava N. (2007). Standard review cold-active microbial lipases: A versatile tool for industrial applications. Biotechnol. Mol. Biol. Rev..

[18] Ollis D.L., Cheah E., Cygler M., Dijkstra B., Frolow F., Franken S.M., Harel M., Remington S.J., Silman I., Schrag J. (1992). The α/β hydrolase fold. Protein Eng..

[19] Casas-Godoy L., Gasteazoro F., Duquesne S., Bordes F., Marty A., Sandoval G. (2018). Lipases: An Overview. Methods Mol. Biol..

[20] Kim T.D. (2017). Bacterial hormone-sensitive lipases (bHSLs): Emerging enzymes for biotechnological applications. J. Microbiol. Biotechnol..

[21] Arpigny J., Jaeger K. (1999). Bacterial lipolytic enzymes: Classification and properties. Biochem. J..

[22] Petrovskaya L.E., Novototskaya-Vlasova K.A., Gapizov S.S., Spirina E.V., Durdenko E.V., Rivkina E.M. (2017). New member of the hormone-sensitive lipase family from the permafrost microbial community. Bioengineered.

[23] Poole L.B. (2015). The basics of thiols and cysteines in redox biology and chemistry. Free Radic. Biol. Med..

[24] Davies M.J. (2005). The oxidative environment and protein damage. Biochim. Biophys. Acta.

[25] Niesen F.H., Berglund H., Vedadi M. (2007). The use of differential scanning fluorimetry to detect ligand interactions that promote protein stability. Nat. Protoc..

[26] Maksimov E., Moldenhauer M., Shirshin E., Parshina E., Sluchanko N., Klementiev K., Tsoraev G., Tavraz N., Willoweit M., Schmitt F.-J. (2016). A comparative study of three signaling forms of the orange carotenoid protein. Photosynth. Res..

[27] Li P.Y., Chen X.L., Ji P., Li C.Y., Wang P., Zhang Y., Xie B.B., Qin Q.L., Su H.N., Zhou B.C. (2015). Interdomain hydrophobic interactions modulate the thermostability of microbial esterases from the hormone-sensitive lipase family. J. Biol. Chem..

[28] Dou S., Kong X.D., Ma B.D., Chen Q., Zhang J., Zhou J., Xu J.H. (2014). Crystal structures of *Pseudomonas putida* esterase reveal the functional role of residues 187 and 287 in substrate binding and chiral recognition. Biochem. Biophys. Res. Commun..

[29] De Simone G., Galdiero S., Manco G., Lang D., Rossi M., Pedone C. (2000). A snapshot of a transition state analogue of a novel thermophilic esterase belonging to the subfamily of mammalian hormone-sensitive lipase. J. Mol. Biol..

[30] Hawe A., Sutter M., Jiskoot W. (2008). Extrinsic Fluorescent Dyes as Tools for Protein Characterization. Pharm. Res..

[31] Joseph B., Ramteke P.W., Thomas G. (2008). Cold active microbial lipases: Some hot issues and recent developments. Biotechnol. Adv..

[32] Zale S.E., Klibanov A.M. (1983). On the role of reversible denaturation (unfolding) in the irreversible thermal inactivation of enzymes. Biotechnol. Bioeng..

[33] Markossian K., Kurganov B. (2004). Protein folding, misfolding, and aggregation. Formation of inclusion bodies and aggresomes. Biochemistry (Moscow).

[34] Finkelstein A.V., Shakhnovich E.I. (1989). Theory of cooperative transitions in protein molecules. II. Phase diagram for a protein molecule in solution. Biopolymers.

[35] Palomo J.M., Fuentes M., Fernández-Lorente G., Mateo C., Guisan J.M., Fernández-Lafuente R. (2003). General Trend of Lipase to Self-Assemble Giving Bimolecular Aggregates Greatly Modifies the Enzyme Functionality. Biomacromolecules.

[36] Jain R., Pandey A., Pasupuleti M., Pande V. (2017). Prolonged Production and Aggregation Complexity of Cold-Active Lipase from *Pseudomonas proteolytica* (GBPI_Hb61) Isolated from Cold Desert Himalaya. Mol. Biotechnol..

[37] Arpigny J.L., Feller G., Gerday C. (1993). Cloning, sequence and structural features of a lipase from the antarctic facultative psychrophile *Psychrobacter immobilis* B10. Biochim. Biophys. Acta Gene Struct. Expr..

[38] Bordes F., Tarquis L., Nicaud J.-M., Marty A. (2011). Isolation of a thermostable variant of Lip2 lipase from *Yarrowia lipolytica* by directed evolution and deeper insight into the denaturation mechanisms involved. J. Biotechnol..

[39] Nam K.-H., Park S.-H., Lee W.-H., Hwang K.-Y. (2010). Biochemical and Structural Analysis of Hormone-sensitive Lipase Homolog EstE7: Insight into the Stabilized Dimerization of HSL-Homolog Proteins. Bull. Kor. Chem. Soc..

[40] Volkin D.B., Klibanov A.M. (1987). Thermal destruction processes in proteins involving cystine residues. J. Biol. Chem..

[41] Slavica A., Dib I., Nidetzky B. (2005). Single-Site Oxidation, Cysteine 108 to Cysteine Sulfinic Acid, in D-Amino Acid Oxidase from *Trigonopsis variabilis* and Its Structural and Functional Consequences. Appl. Environ. Microbiol..

[42] Perry L.J., Wetzel R. (1987). The role of cysteine oxidation in the thermal inactivation of T4 lysozyme. Protein Eng..

[43] Suemori A., Iwakura M. (2007). A systematic and comprehensive combinatorial approach to simultaneously improve the activity, reaction specificity, and thermal stability of p-hydroxybenzoate hydroxylase. J. Biol. Chem..

[44] Kumar S., Tsai C.-J., Nussinov R. (2000). Factors enhancing protein thermostability. Protein Eng..

[45] Dominy B.N., Minoux H., Brooks III C.L. (2004). An electrostatic basis for the stability of thermophilic proteins. Proteins: Struct. Funct. Bioinf..

[46] Gromiha M.M., Pathak M.C., Saraboji K., Ortlund E.A., Gaucher E.A. (2013). Hydrophobic environment is a key factor for the stability of thermophilic proteins. Proteins: Struct. Funct. Bioinf..

[47] Vogt G., Woell S., Argos P. (1997). Protein thermal stability, hydrogen bonds, and ion pairs. J. Mol. Biol..

[48] Mukaiyama A., Takano K., Sen S., Nilsson L. (2012). Delineation of the Conformational Thermostability of Hyperthermophilic Proteins Based on Structural and Biophysical Analyses. Thermostable Proteins.

[49] Timucin E., Sezerman O.U. (2013). The conserved lid tryptophan, W211, potentiates thermostability and thermoactivity in bacterial thermoalkalophilic lipases. PLoS ONE.

[50] Zhu K., Jutila A., Tuominen E.K., Patkar S.A., Svendsen A., Kinnunen P.K. (2001). Impact of the tryptophan residues of *Humicola lanuginosa* lipase on its thermal stability. Biochim. Biophys. Acta Protein Struct. Mol. Enzymol..

[51] Dror A., Shemesh E., Dayan N., Fishman A. (2014). Protein engineering by random mutagenesis and structure-guided consensus of *Geobacillus stearothermophilus* lipase T6 for enhanced stability in methanol. Appl. Environ. Microbiol..

[52] Santarossa G., Lafranconi P.G., Alquati C., DeGioia L., Alberghina L., Fantucci P., Lotti M. (2005). Mutations in the “lid” region affect chain length specificity and thermostability of a *Pseudomonas fragi* lipase. FEBS Lett..

[53] Abraham T., Abraham T., Pil Pack S., Je Yoo Y. (2005). Stabilization of *Bacillus subtilis* Lipase A by increasing the residual packing. Biocatal. Biotransform..

[54] D’Amico S., Gerday C., Feller G. (2003). Temperature Adaptation of Proteins: Engineering Mesophilic-like Activity and Stability in a Cold-adapted α-Amylase. J. Mol. Biol..

[55] Salwoom L., Rahman R.A., Zaliha R.N., Salleh A.B., Convey P., Ali M., Shukuri M. (2019). New recombinant cold-adapted and organic solvent tolerant lipase from psychrophilic *Pseudomonas sp*. LSK25, isolated from Signy Island Antarctica. Int. J. Mol. Sci..

[56] Li P.Y., Zhang Y., Xie B.B., Zhang Y.Q., Hao J., Wang Y., Wang P., Li C.Y., Qin Q.L., Zhang X.Y. (2017). Structural and Mechanistic Insights into the Improvement of the Halotolerance of a Marine Microbial Esterase by Increasing Intra- and Interdomain Hydrophobic Interactions. Appl. Environ. Microbiol..

[57] Li B., Yang G., Wu L., Feng Y. (2012). Role of the NC-loop in catalytic activity and stability in lipase from *Fervidobacterium changbaicum*. PLoS ONE.

