# Supplementary material for: Thermal Inactivation of a Cold-Active Esterase PMGL3 Isolated from the Permafrost Metagenomic Library
Source: Biomolecules. 2019 Dec 16;9(12):880. doi: 10.3390/biom9120880 (PMC6995580; doi:10.3390/biom9120880)
Supplement: Supplementary file 1 [file biomolecules-09-00880-s001.pdf]

Supplementary Material to the manuscript **“Thermal inactivation of a cold-active esterase PMGL3 isolated from the permafrost metagenomic library”** by M.V. Kryukova et al.

Table S1. Content of the secondary structure elements in PMGL3 and mutant variants obtained by CD spectroscopy at different temperatures.

Protein	**Temperature, °C**	**α-helix,** %	**ß-sheet,** %	**unregular,** %
**PMGL3**	20	31.3	16.6	52.1
	30	30.6	18.1	51.3
	40	27.3	20.5	52.1
	50	15.6	27.9	56.5
**C49V**	20	32.8	17.8	49.5
	30	31.0	17.5	51.6
	40	27.8	19.2	53.0
	50	17.6	26.9	55.5
**C207F**	20	29.6	22.7	47.7
	30	28.2	23.7	48.0
	40	27.4	22.7	49.9
	50	16.2	31.4	52.4

Figure S1. Location of cysteine residues and residues belonging to the catalytic triad in the PMGL3 molecule. Ribbon presentations are colored according to the predicted secondary structure of the protein; green and blue elements indicate α-helices and β-sheets, respectively. A structure model of PMGL3 was generated using SWISS-MODEL server based on PDB entry 5GMR as a template and drawn using Swiss-PdbViewer.

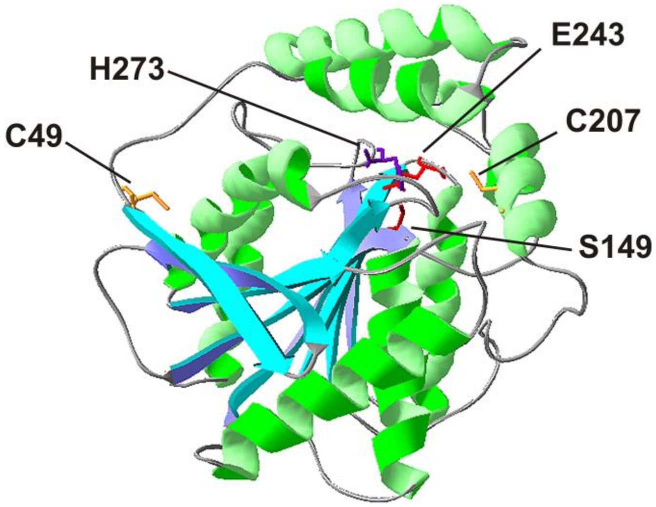

Figure S2. Characteristic fluorescence decay kinetics of bis-ANS in the buffer and in protein solutions of PMGL3 and mutant variants at 25°C.

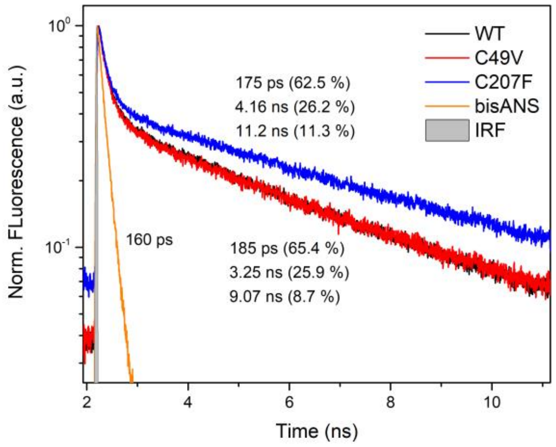

Figure S3. Melting of PMGL3 and mutant variants as revealed by bis-ANS fluorescence (**A**) and intrinsic Trp fluorescence (**B**).

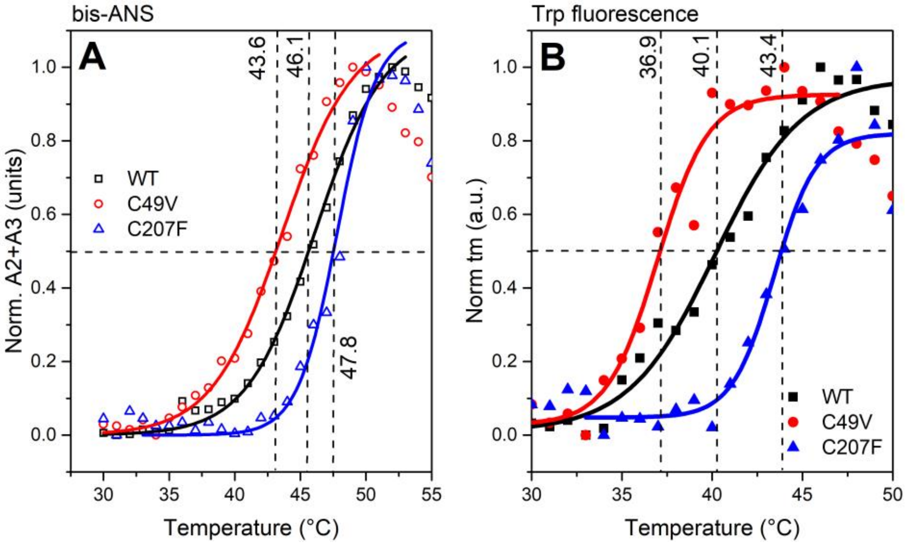

bis-ANS
A
43.6
46.1
47.8
Norm. A2+A3 (units)
WT
C49V
C207F
Temperature (°C)
Trp fluorescence
B
36.9
40.1
43.4
Norm tm (a.u.)
WT
C49V
C207F
Temperature (°C)
